# Multiple micronutrients fortified salt: consumers’ acceptability survey, Tanzania

**DOI:** 10.1186/s12889-023-15000-7

**Published:** 2023-01-11

**Authors:** Paschal Mdoe, Venkatesh Mannar, Bernadetha Paulo, Godfrey Guga, Castory Mushi, Caroline Kimathi, John Paschal, Samwel Jatosh, Vincent Assey, Fatma Abdallah, Estomih Mduma, Levente Diosady

**Affiliations:** 1 Haydom Lutheran Hospital, P.O. Box 9000 , Manyara Haydom, Tanzania; 2 University of Toronto, Toronto, Canada; 3 Food and Nutrition Center, Dar es Salaam, Tanzania

**Keywords:** Salt fortification, Quadruple fortified salt, Double fortified salt, Iodized salt

## Abstract

**Background:**

Food fortification with micronutrients is an insufficiently used technology in developing countries. Salt is consumed in small, constant daily amounts by most people globally. Salt has been instrumental in delivering iodine to a wide population globally through fortification. There is a proven effective technology for fortifying iodinated salt with iron, folate, and Vitamin B12. Findings have shown that both Double (Iodine and iron) fortified salt (DFS) and quadruple (iron, iodine, folate, and vitamin B12) fortified salt (QFS) are effective in raising hemoglobin levels.

**Aim:**

To assess the acceptability and gauge consumers’ willingness to use double-fortified and quadruple-fortified salt formulations.

**Methods:**

We conducted an observational study involving 300 households at Haydom Lutheran Hospital catchment area in Northern rural Tanzania between October 2021 and April 2022. Each household was supplied with one type of salt (iodized salt (IS), DFS or QFS) for cooking common family dishes for one week. Thereafter, at least two adult members of the family who used the dishes cooked with study salt were interviewed using the adopted 5-point Hedonic scale.

**Results:**

A total of 899 individuals were interviewed after using study salt for one week: 286 IS, 305 DFS, and 308 QFS. The overall acceptability for the salts was QFS (82%), DFS (78%), and IS (79%). The mean sensory (taste, color and appearance) scores of the QFS (1.7) and DFS (1.7) were comparable to standard iodized salt (1.6).

**Conclusion:**

Quadruple-fortified salt and double-fortified salt are equally acceptable and have similar sensory scores as standard iodized salt when used to cook commonly eaten dishes in the study population.

## Background

Food fortification with micronutrients has been practiced as a way of delivering essential vitamins and minerals to the population in developed countries for decades [1, 2]. However, food fortification is insufficiently used in developing countries. For an effective food fortification program, there is a need to identify a suitable vehicle and the process must be properly managed to be cost-effective [1, 3]. Salt is consumed in small, constant daily amounts by almost everyone and is often considered an ideal vehicle for vitamins and minerals [4]. The success of salt iodization initiatives around the world has been well documented, demonstrating significant reductions in the prevalence of Iodine Deficiency Disorders in areas where it has been implemented [5–7]. Progress in adding iodine to two-thirds of the world’s household salt has fueled the hopes that the problem of iron deficiency might be similarly tackled through the double fortification of salt (DFS) [8, 9]. Various formulations of DFS have been developed in several countries [10]. DFS based on technology developed at the University of Toronto was successfully tested on a large scale in India [11], and resulted in a significant increase in the level of iron to those who consumed [12]. In Tanzania, double fortification of salt is now being tested and can be a viable technology for adding iron to iodized salt in a stable and cost-effective manner to overcome two major public health problems.

The latest research in this area has focused on the development of multiple micronutrient combinations in fortified salt e.g. with iodine, iron, folic acid, and vitamin B12. An initial test on QFS has been completed in Tanzania [13] and tests are underway in India. A recent sensory study of multiple-fortified salt found no obvious sensory differences for foods prepared with either of the IS, DFS, or QFS. Furthermore, the results of the effectiveness trial showed that QFS is superior to DFS and IS in raising haemoglobin level of anemic women. Yadav et al. 2019 meta-analysis found that DFS is a potentially efficacious strategy for addressing anemia as a public health problem at a population level [14]. However, there has been no published community perception study that assesses acceptability by the general population of multiple fortified salts in daily use. We conducted a community survey to assess the acceptability and gauge consumers’ willingness to use double-fortified and quadruple-fortified salt formulations.

## Methodology

### Study site

The study was conducted within the Haydom Lutheran Hospital catchment area involving 6 villages within a radius of 50 Km. The site was selected based on outstanding experience in community-based research. Furthermore, Haydom Lutheran Hospital has a well-established research infrastructure for conducting community-based research.

### Salt preparation

Double fortified salt was prepared by mixing a ferrous fumarate premix, prepared using the technology developed by the University of Toronto (Li et al.) by JVS Foods Pvt lTd, Jaipur, India, with locally produced commercial iodized salt. The iron content of DFS and QFS was 1000 µg/g. The QFS apart from iodine and iron it also contained folic acid 40 µg/g, and Vitamin B12 2.4 µg/g [13].

### Study design

This was a cross-sectional observational study involving healthy adults aged 18 years and above from villages surrounding the Haydom Lutheran Hospital. The study involved two phases: a preparatory phase and a consumer’s sensory/perception assessment phase.

In the preparatory phase, the list of all villages within a radius of 50 km from Haydom Lutheran Hospital was generated. Six villages were randomly selected and involved in the study. For all the selected villages, a list of households was obtained from the village leader. At least 100 households from each village were randomly selected and approached for consent to participate in the study. For the household that consents, the female heads of these households were interviewed to gather basic household information which included: the number of people eating common food cooked in the household, type of family, and salt use practice. Both the research assistant and the household were not aware of the randomized arm. Thereafter, households were randomly assigned to either Iodized salt, Double (Iodine + Iron) fortified salt or Quadruple (Iodine + Iron + Vitamin B12 + Folic acid) fortified salt. The fortified salt types (IS, DFS, and QFS) were randomly supplied to study households (a household received only one type of the three salt types). The household was allowed to use the study salt for cooking different dishes as they normally use with ordinary salt for one week.

Consumers’ sensory/perception assessment phase followed one week of study salt use. The research assistant visited the household again and enquired about the remaining salt and the salt use over the past one week. Two to three adults were interviewed using a semi-structured questionnaire (5-point Hedonic scale). They were asked to report on the appearance of the food as compared to the experience with ordinarily used salt, color, and taste of the food. A scoring pattern (5-point Hedonic scale) was used for various parameters i.e., color, texture, appearance, taste, and overall acceptability of the cooked dishes. Further, they were interviewed on the stored food if they noticed changes in appearance, color, and taste compared to what they were used to when using their ordinary salt. All the participants were not trained (untrained panelists) and the attributes used in the questionnaire (5-points Hedonic scale) were not shared with them before the interview.

### Data collection and management

Trained research assistants (RA) visited each household to obtain voluntary written informed consent from the head of household. The research assistant interviewed the head of household to collect general household information. The RA supplied the amount of salt type assigned for the household and enough to use for one week. RA asked the household to use the study salt in cooking common dishes. The RA visited the household twice during the week to assess the level of study salt usage. At the end of one week of using the study salt, the RA interviewed the female head of the household and other two adult members regarding their perception on the use of the study salt. All data went through two stages of quality control (QC) before being entered into a database: initial QC was done by RAs at the field, making sure that all information has been correctly entered in the data collection form, the second QC was done by designated QC personnel at the research center

### Data analysis

The cleaned data were analysed using R-Command where the dependent variables were color, appearance and taste and overall acceptability of the cooked dishes. The independent variables were the three types of salt: IS, DFS and QFS.

The data were assessed for normality using Shapiro-Wilk test and Q-Q plot. The normally distributed variables, and the difference in mean scores were tested using the Kruskal Wallis test. Differences were significant when the p-value was equal or less than 0.05.

## Results

Between October 2021 and April 2022, a total of 300 households from 6 villages were visited and consented to participate in the study. Mean age of the female head of the household visited was 38 years, and each household had an average of 7 people. Over 90% of the visited households obtained their salt from the nearby shops and almost three-quarters of them used iodized salt for cooking. However, about a quarter was not aware of whether they used iodized salt or not (Table 1).

**Table 1:** Demographic characteristics and basic information of the participants

		IS	DFS	QFS
Households used salt (N)		100	102	98
Age (Years) of female head of the Household (Median (Q1, Q2)		37 (30, 48)	39 (29, 48)	37 (30, 48)
People living in the household (mean (Q1, Q2)		6.6 (5, 8)	6.9 (5, 9)	6.5 (5, 8)
Source of salt commonly used in a household	Shop (Iodized salt)	91 (91%)	98 (96%)	89 (90.8%)
	Nearby salt farm (non-iodized salt)	9 (9%)	4 (4%)	9 (9.2%)
Type of salt normally used in a household	Iodised	76 (76%)	79 (77.5%)	80 (81.6%)
	Don’t know	24 (24%)	23 (22.5%)	18 (18.4%)
Total individuals interviewed after salt use	Female	237 (82.9%)	240 (78.9%)	260 (84.4%)
	Male	49 (17.1%)	65 (21.1%)	48 (15.6%)

**Fig. 1:**
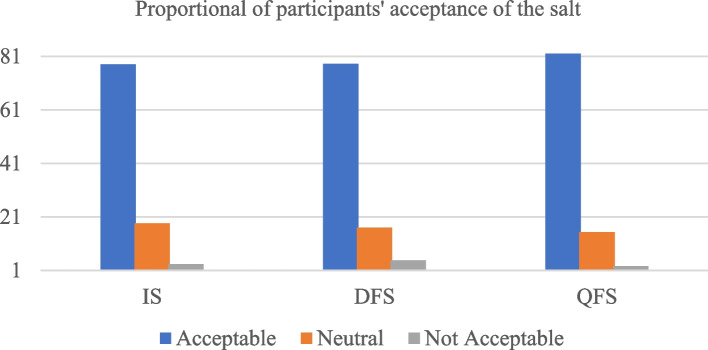
Participants’ acceptance level of different salt types

The salt acceptability survey showed that over 78% of all participants accepted all types of salt equally. Very few, less than 5% of the respondents reported to not accept any type of the three salt types (Fig. 1).

The descriptive analysis (Table 1) shows that all three types of salt (i.e., IS, DFS and QFS) were equally acceptable among the participants interviewed. Furthermore, the sensory assessment, that is the appearance of food, its color, and taste was equally gauged by the three study salts. Therefore, there are not statistically significant differences in the levels of acceptability, food appearance, color of the food, and taste of the food among the salt groups (IS, DFS and QFS) (Table 2).

**Table 2:** The sensory and acceptability characteristics of three different salt types

		N	Mean	Std. Deviation	Std. Error	95% Confidence Interval for Mean		Minimum	Maximum
						Lower Bound	Upper Bound		
Appearance	IS	286	1.6678	0.96144	0.05685	1.5559	1.7797	1.00	5.00
	DFS	305	1.7246	0.96802	0.05543	1.6155	1.8337	1.00	5.00
	QFS	308	1.6591	0.97727	0.05569	1.5495	1.7687	1.00	5.00
	Total	899	1.6841	0.96848	0.03230	1.6207	1.7475	1.00	5.00
Colour	IS	286	1.7413	1.01368	0.05994	1.6233	1.8592	1.00	5.00
	DFS	305	1.7574	0.98014	0.05612	1.6469	1.8678	1.00	5.00
	QFS	308	1.7013	0.97279	0.05543	1.5922	1.8104	1.00	5.00
	Total	899	1.7330	0.98763	0.03294	1.6684	1.7977	1.00	5.00
Taste	IS	286	1.5524	0.93041	0.05502	1.4442	1.6607	1.00	5.00
	DFS	305	1.6426	1.00663	0.05764	1.5292	1.7560	1.00	5.00
	QFS	308	1.6201	0.99273	0.05657	1.5088	1.7314	1.00	5.00
	Total	899	1.6062	0.97782	0.03261	1.5422	1.6702	1.00	5.00
Acceptability	IS	286	1.4825	0.88934	0.05259	1.3790	1.5860	1.00	5.00
	DFS	305	1.5738	0.96755	0.05540	1.4648	1.6828	1.00	5.00
	QFS	306	1.5523	0.95413	0.05454	1.4450	1.6596	1.00	5.00
	Total	897	1.5373	0.93839	0.03133	1.4759	1.5988	1.00	5.00

The ANOVA tests show no difference between salt types in terms of general acceptability, food appearance, food color, and food taste when IS, DFS, or QFS was used for cooking (Table 3).

**Table 3:** The comparison of sensory characteristics between salt types

ANOVA						
		Sum of Squares	df	Mean Square	F	Sig.
Appearance	Between Groups	0.768	2	0.384	0.409	0.664
	Within Groups	841.5	896	0.939		
	Total	842.2	898			
Colour	Between Groups	0.510	2	0.255	0.261	0.770
	Within Groups	875.4	896	0.977		
	Total	875.9	898			
Taste	Between Groups	1.291	2	0.645	0.674	0.510
	Within Groups	857.3	896	0.957		
	Total	858.6	898			
Acceptability	Between Groups	1.333	2	0.666	0.756	0.470
	Within Groups	787.6	894	0.881		
	Total	788.9	896			

## Discussion

This report presents the findings of an acceptability study. The overall acceptability and the sensory score of the quadruple (Iodine, iron, folate, and vitamin B12) fortified salt, and double (iodine and iron) fortified salt were compared to standard iodized salt. Overall results show that both quadruple (Iodine, iron, folate, and vitamin B12) fortified salt, and double (iodine and iron) fortified salt are equally acceptable as standard iodized salt among the communities in rural Tanzania. Furthermore, in terms of food appearance, color and taste, participants found no difference between meals prepared using QFS, DFS and standard IS.

Salt iodization has been implemented in nearly all countries worldwide, and two thirds of the world’s population is now consuming iodized salt. Almost 90% of households worldwide use iodized salt [15]. The situation in the sampled areas of Tanzania is similar. This study found that over 90% of all household’s heads indicated that they procure iodized salt from nearby shops. Tanzania has a well-established mechanism for fortifying salt with iodine thus iodization of table salt is a global health success. The success of salt iodization presents an opportunity to build on existing resources for addressing other micronutrient deficiencies.

The University of Toronto developed technology for producing premixes of iron, iodine, folate, and Vitamin B12 which is readily admixed to salt to produce fortified salt. We used this technology to develop DFS and QFS which have been tested in a clinical trial showing impressive results in improving the Haemoglobin level of women of reproductive age and improving the micronutrient level of the consumers. This acceptability study shows that all households that used QFS and DFS for cooking common dishes in their households found no difference from commonly used salt (IS). They found that the food’s appearance, color, and the taste was similar to that obtained with iodized salt.

## Conclusion

The findings of this study show that consumers accept Double (iron and iodine) fortified salt and Quadruple (iron, iodine, vitamin B12, and folate) fortified salt equally as they accept iodized salt for cooking common dishes in their households. Furthermore, consumers found no difference in terms of appearance of the food, color of the food, and taste of the food when using either DFS, QFS or IS.

## Data Availability

Data are available from the first author (PM) upon request.
